# Development of Fermented Shrimp Shell Product with Hypoglycemic and Hypolipidemic Effects on Diabetic Rats

**DOI:** 10.3390/metabo12080695

**Published:** 2022-07-27

**Authors:** Chung-Hsiung Huang, Chih-Heng Lin, Hsiao-Han Huang, Guo-Jane Tsai

**Affiliations:** 1 Department of Food Science, National Taiwan Ocean University, Keelung 20224, Taiwan; huangch@mail.ntou.edu.tw (C.-H.H.); cloudkumo@gmail.com (C.-H.L.); ki5566ss1@gmail.com (H.-H.H.); 2 Center for Marine Bioscience and Biotechnology, National Taiwan Ocean University, Keelung 20224, Taiwan

**Keywords:** astaxanthin, hypoglycemic, hypolipidemic, mixed strain fermentation, shrimp shell

## Abstract

In 2020, approximately 9.3 billion tons of crustaceans were consumed, and 45–48% of shrimp shell (SS) by-products were discarded as waste. In this study, the SS of *Litopenaeus vannamei* was fermented by *Lactobacillus plantarum* LV33204, *Stenotrophomonas maltophilia* LV2122 (strong proteolytic activity), and *Aeromonas dhakensis* LV1111 (chitin-degrading activity), and the optimal fermentation conditions of liquid-fermented SS was established. Contents of total peptide, astaxanthin, and total phenolic content of the fermented SS were significantly higher than that of unfermented SS. In the presence of fermented SS, glucose uptake and insulin resistance of TNF-α-stimulated FL83B hepatocytes were markedly improved. Furthermore, daily oral supplement of fermented SS to streptozotocin (STZ)/nicotinamide (NA)-induced diabetic rats for 7 weeks significantly reduced plasma glucose and insulin resistance. Meanwhile, ingestion of fermented SS might enhance hepatic catabolism of glucose by increasing hexokinase and glucose-6-phosphate dehydrogenase activity and decreasing glucose-6-phosphatase activity. In addition, the fermented SS downregulated plasma total cholesterol (TG), triglycerides (TCs), low-density lipoprotein cholesterol (LDL-C), liver TG, and TC and lipid peroxidation levels in diabetic rats. In conclusion, a biorefinery process for waste SS was established through mixed strain fermentation. The in vitro and in vivo data reveal that the fermented SS is a promising functional food for the management of diabetic hyperglycemia and hyperlipidemia.

## 1. Introduction

About 90% of diagnosed cases of diabetes are type 2 diabetes, which is a long-term metabolic disorder that usually results from obesity and physical inactivity. Insulin resistance, a condition linked to type 2 diabetes, is the contributor to hyperglycemia and related complications, including cardiovascular diseases, brain attack, retinopathy, renal failure, and ischemic foot with gangrene [1,2]. The fasting plasma glucose test, oral glucose tolerance test (OGTT), and homoeostasis model assessment of insulin resistance (HOMA-IR) are the common and simple methods for the diagnosis of type 2 diabetes [3]. Thus far, loss of body weight, healthy diets, regular exercise, medication or insulin injection, and blood sugar monitoring are the major approaches to manage type 2 diabetes [2]. However, these approaches are only designed to delay the development or pathogenesis of complications. In addition to insulin resistance, endogenous glucose production is also a factor leading to hyperglycemia in patients with type 2 diabetes. Hepatic glucose metabolic enzymes play an important role in endogenous glucose homoeostasis [4]. For example, glucose-6-phosphatase and hexokinase are crucial for endogenous glucose production, and it is suggested that the ratio of glucose-6-phosphatase/hexokinase activities is correlated with the severity of hyperglycemia [4]. Moreover, glucose-6-phosphate dehydrogenase is the key enzyme triggering the pentose phosphate pathway, and a close association between diabetes and a glucose-6-phosphate dehydrogenase deficiency has been substantiated [5]. On the other hand, lipid metabolism is considered equally critical to glucose metabolism in the progression of diabetes. Visceral fat is considered a major contributor not only to dyslipidemia, but also to insulin resistance in type 2 diabetes [6]. Dyslipidemia is recognized as a prominent risk factor for cardiovascular disease, and it can be early detected to prevent the occurrence of disease [7]. Dyslipidemia, including diminished levels of high-density lipoprotein cholesterol (HDL-C) and raised levels of low-density lipoprotein cholesterol (LDL-C) and triglycerides (TGs), influences about 50% of patients with type 2 diabetes. High generation and low clearance of very-low-density lipoproteins (VLDL) by the liver, eliciting their conversion to LDLs, are critical for the progression of dyslipidemia [8]. Furthermore, oxidative stress and lipid peroxidation play crucial roles in the development of diabetes and the related complications [9]. Although appropriate diet, exercise, and weight management can alleviate diabetic hyperglycemia and dyslipidemia, natural antioxidants or dietary supplements are urgently sought to improve diabetes symptoms [10].

Industrial processing of seafood faces environmental and processing plant problems in major fish-producing countries. About 45–60% of waste is produced from shrimp processing, mainly consisting of shrimp heads and shells [11]. The shrimp waste is rich in proteins, minerals, and chitin. For example, the white shrimp (*Litopenaeus vannamei*) shell contains 32% chitin, 35% crude protein, and 28% minerals. In addition, it also contains biologically active substances such as astaxanthin, enzymes, and peptides [12,13,14]. However, only about 5% of shrimp waste is utilized in animal feed. Reutilization of these shrimp wastes is beneficial to decrease the accumulation of waste from processing plants. The development of value-added shrimp waste functional products not only improves the economics of shrimp processing, but also reduces the environmental pollution of shrimp waste [15].

Several studies have reported the beneficial effects of components derived from SS on improving diabetes. In animal models of diabetes, administration of astaxanthin improved insulin resistance and insulin secretion, reduced hyperglycemia, and attenuated retinopathy, nephropathy, and neuropathy [16]. Shrimp chitosan has beneficial activities that protect and proliferate pancreatic beta cells, reduce hyperglycemia, and prevent impaired lipid metabolism in diabetes [17]. The current research on SS microbial fermentation mainly focuses on the removal of proteins and minerals bound to chitin or astaxanthin to recover chitin or astaxanthin in shrimp shells, especially the recovery of chitin or its derivatives. Either a single strain of *Lactobacillus*, *Streptococcus*, and *Bacillus* species, or mixed strains of *Lactobacillus* sp. plus *Teredinobacter turnirae* or *Serratia marcescens* B742 have been used to separate SS chitin/chitosan [18,19,20,21]. However, research pertaining to the impact of fermented SS on metabolism disorders is not yet available. In this study, we established the process of fermentation by mixed strains of functional microbes to obtain fermented SS with markedly higher contents of peptides, astaxanthin, and total phenolic content. Further, the effect of fermented SS on ameliorating insulin resistance was examined in FL83B hepatocytes. The hypoglycemic and hypolipidemic effects of fermented SS was explored in a rat model of STZ/NA-induced diabetes. Subsequently, the effects of fermented SS on modulating hepatic enzymes for glucose metabolism and attenuating lipid peroxidation were illustrated.

## 2. Results

### 2.1. Changes in the Contents of Components within Fermented SS

**Figure 1:**
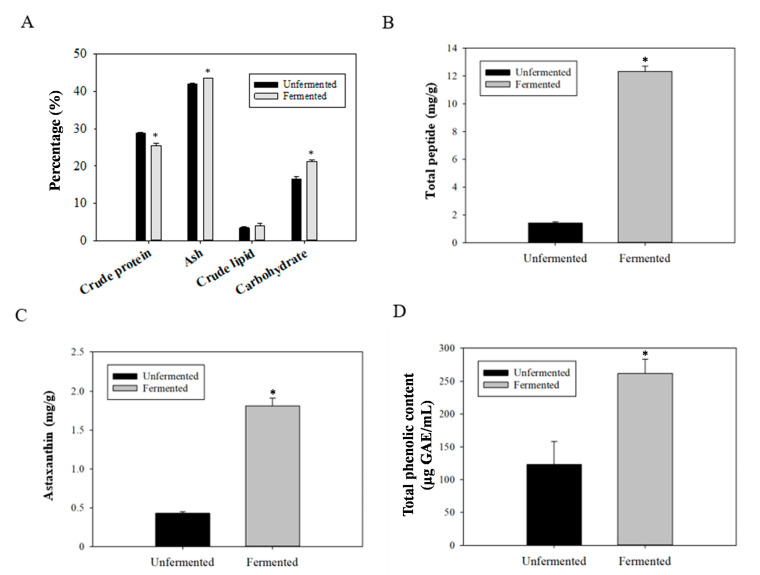
(**A**) Proximate composition, and contents of (**B**) total peptides, (**C**) astaxanthin, and (**D**) total phenolic content in the lyophilized products of unfermented and fermented shrimp shell. Results are expressed as mean ± S.D. (N = 3). * *p* < 0.05 compared with unfermented control.

A previous study reported that SS is abundant in bioactive and antioxidant phenolic compounds [22]. The SS structure from outside to inside is divided into the epicuticle, exocuticle, and endocuticles. Chitin is found mainly in the endocuticles surrounded by a layer of protein. The exocuticle consists of chitin and minerals, the epicuticle consists of proteins and minerals, and astaxanthin is bound to protein [23]. Therefore, three strains isolated from Litopenaeus vannamei (white shrimp), including *Lactobacillus plantarum* LV33204, *Stenotrophomonas maltophilia* LV2122 (strong proteolytic activity), and *Aeromonas dhakensis* LV1111 (chitin-degrading activity), were used together for the fermentation of Litopenaeus vannamei SS to dissociate and degrade chitin and protein. Compared with the proximate composition of unfermented SS, the crude protein content of fermented SS was significantly reduced, whereas the ash and carbohydrate contents were significantly increased (Figure 1A). The contents of total peptides and astaxanthin in SS were increased after fermentation (Figure 1B,C). Additionally, the total phenolic content in the supernatant of fermented SS was greatly increased (Figure 1D). These results demonstrate that the mixed strains could hydrolyze proteins as a nutrient source and release phenols and astaxanthin during fermentation.

As shown in Table 1, the content of total free amino acids in fermented SS was greatly increased compared with that in unfermented SS. The essential amino acids including threonine, valine, methionine, isoleucine, leucine, phenylalanine, and histidine increased from 11.14 mg/100 g in unfermented SS to 122.61 mg/100 g in fermented SS, indicating that fermented SS can be potentially used as a nutritional supplement or health food. Notably, the content of amino acids that impart sweetness to SS, such as alanine, proline, and glycine, was also increased after fermentation, showing that fermentation with these mixed strains may improve the flavor of SS waste [24].

### 2.2. Effect of Fermented SS on Upregulating Glucose Uptake of FL83B Hepatocytes

**Figure 2:**
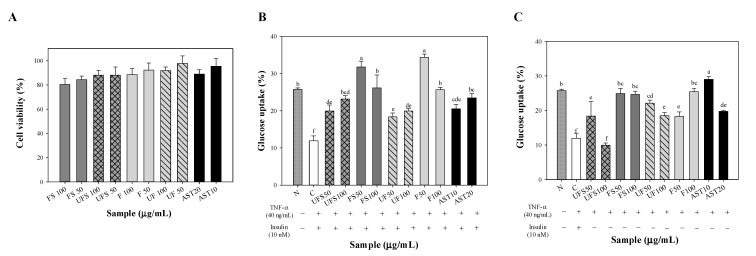
The effect of unfermented and fermented shrimp shell on (**A**) cell viability and glucose uptake of insulin resistant FL83B hepatocytes treated (**B**) with or (**C**) without insulin. Results are expressed as mean ± S.D. (N = 3). Different letters indicate significant difference between each group. UFS50, UFS100, FS50, FS100 mean the culture filtrates of unfermented SS and fermented SS at dosages of 50 and 100 g/mL. UF50, UF100, F50, F100 mean the methanol extract of the dry powders of unfermented SS and fermented SS at dosages of 50 and 100 g/mL. AST10 and AST20 mean astaxanthin standard at dosages of 10 and 20 g/mL.

As hepatocytes play a critical role in glucose metabolism, the influence of fermented SS on glucose uptake of insulin-resistant FL83B hepatocytes was further evaluated. After 24 h of incubation with unfermented or fermented SS supernatant (50 or 100 μg/mL), unfermented or fermented SS methanol extract (50 or 100 μg/mL), or astaxanthin (AST, 10 or 20 μg/mL), the viability of FL83B cells was greater than 80% (Figure 2A). Pursuant to the methods reported in the previous studies and ISO 10993-5, percentages of cell viability above 80% are considered to be non-cytotoxic [25,26]. Accordingly, theses results indicate that the employed concentrations of samples were not toxic to FL83B cells. Since TNF-α could induce insulin resistance [27], the effect of fermented SS on glucose uptake was investigated in TNF-α-induced FL83B cells in the presence of insulin. Although both unfermented SS supernatant and unfermented SS methanolic extract upregulated glucose uptake in FL83B cells, fermented SS samples promoted glucose uptake more efficiently than unfermented SS samples (Figure 2B). Since lack of insulin leads to type 1 diabetes, the effect of fermented SS on glucose uptake was also investigated in FL83B cells in the absence of insulin. Interestingly, fermented SS supernatants and fermented SS methanol extracts markedly promoted glucose uptake (Figure 2C).

### 2.3. Influence of Fermented SS on the General Physiological Parameters of Diabetic Rats

**Figure 3:**
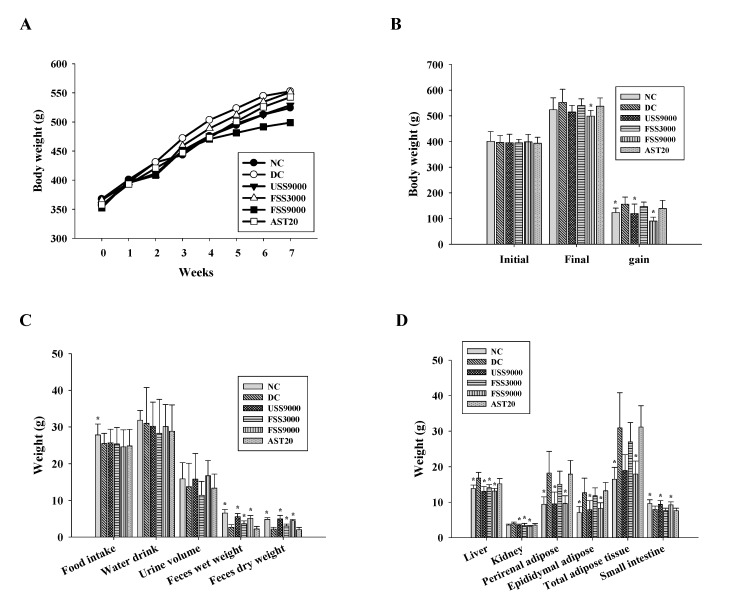
The changes of (**A**) average body weight, (**B**) gain of body weight, (**C**) average food intake, drinking water intake, urine volume, feces weights, and (**D**) weights of organs and adipose tissues of rats fed with various diets for 7 weeks. Result are expressed as mean ± SD for each group of rats (N = 6). * *p* < 0.05 compared with diabetic control (DC).

Based on the above results showing that fermented SS has an upregulatory effect on glucose uptake, the rat model of diabetes was employed to explore the in vivo effect of fermented SS on improving diabetes. The results of a previous study indicated beneficial effects of estrogen on type 2 diabetes [28]. In contrast, Vital et al. demonstrated that females were less sensitive to insulin and more susceptible to the rapid development of a more severe form of diabetes [29]. To avoid the interference from estrogen, male rats were employed in this study. Because streptozotocin (STZ) selectively destroys pancreatic beta cells, whereas nicotinamide (NA) decreases the damage caused by STZ, STZ/NA creates a state of partial insulin deficiency similar to what occurs in type 2 diabetes [30]. Therefore, the rat model of diabetes was induced by STZ/NA as described in the Materials and Methods section. Because fermented SS was designed to be developed into functional foods, the samples were administrated to rats by oral route. As shown in Figure 1, fermented SS comprised higher levels of hypoglycemic compounds, and the content of astaxanthin was 1.81 mg/g. To compare the potency of fermented SS to the astaxanthin control (20 mg/kg BW), the dose of fermented SS containing astaxanthin with the content approaching that of the astaxanthin control was employed. Therefore, FSS9000 (9000 mg/kg BW), containing astaxanthin at the dose of 16.29 mg/kg BW, was designed as the high-dose treatment, and FSS3000 (3000 mg/kg BW) was designed as the low-dose treatment. As shown in Figure 3A,B, the gaining of body weight of rats fed with unfermented SS and fermented SS was significantly decreased compared to that of rats in the DC control group. Concordantly, the feces weight of rats fed with unfermented SS and fermented SS was also increased (Figure 3C). Among the treatments, FSS9000 was the most potent one that lowered the weights of liver, kidney, and adipose tissues (Figure 3D). In contrast, unfermented SS and fermented SS treatments reversed the loss of small intestinal weight in diabetic rats (Figure 3D). Since previous studies also reported the inhibitory activity of chitosan on lipogenesis [31], it was suggested that fermented SS has the potential to exert anti-lipogenic and anti-obesity effects.

### 2.4. Oral Administation with Fermented SS Alleviated Hyperglycemia in Diabetic Rats

**Figure 4:**
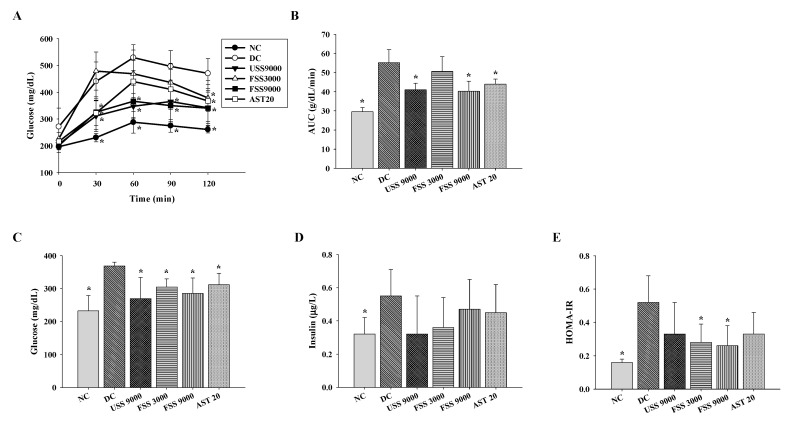
The plasma concentration of (**A**) glucose, (**B**) glucose area under the curve (AUC) during oral glucose tolerance test, and the levels of (**C**) fasting plasma glucose, (**D**) insulin, and (**E**) HOMA-IR of diabetic rats fed with various diets for 7 weeks. Results are expressed as mean ± SD for each group of rats (N = 6). * *p* < 0.05 compared with diabetic control (DC).

**Figure 5:**
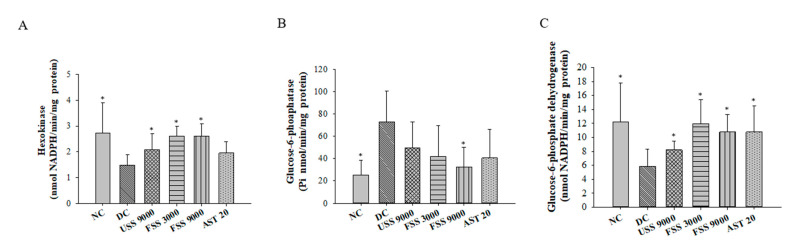
The activities of hepatic glucose metabolic enzymes (**A**) hexokinase, (**B**) glucose-6-phosphatase, and (**C**) glucose-6-phosphate dehydrogenase of rats fed with various diets for 7 weeks. Results are expressed as mean ± SD for each group of rats (N = 6). * *p* < 0.05 compared with diabetic control (DC).

To investigate the in vivo hypoglycemic activity of fermented SS, the OGTT test was conducted in STZ/NA-induced diabetic rats fed with unfermented SS, fermented SS, or astaxanthin daily for 7 weeks. Treatment with unfermented SS (9000 mg/kg BW), fermented SS (9000 mg/kg BW), or astaxanthin (20 mg/kg BW) reduced the plasma concentration of glucose and the area under the curve (AUC) of plasma glucose (Figure 4A,B). In fasting rats, the plasma levels of glucose and insulin of the DC group were significantly higher than that of the NC group (Figure 4C,D). In parallel, unfermented SS, fermented SS, and astaxanthin lowered the plasma concentration of glucose (Figure 4C). Since the homeostasis model assessment of insulin resistance (HOMA-IR) is a simple and useful method for evaluating insulin sensitivity [32], the HOMA-IR was employed in the current study. As shown in Figure 4E, fermented SS and astaxanthin were more potent at lowering the HOMA-IR, indicating the beneficial effect of fermented SS against insulin resistance. However, further investigation is required to evaluate whether fermented SS could improve type 1 diabetes in vivo. On the other hand, the activities of crucial glucose metabolic enzymes in the liver were determined to illustrate the potential mechanism of the fermented SS-mediated hypoglycemic effect. Compared to those in the normal rats, the levels of hexokinase and glucose-6-phosphate dehydrogenase were diminished, and the level of glucose-6-phosphatase was elevated in diabetic control rats (Figure 4). However, unfermented SS and fermented SS treatment could reverse the diminished levels of hexokinase and glucose-6-phosphate dehydrogenase (Figure 5A,C). Notably, treatment with fermented SS at 9000 mg/kg BW significantly downregulated the hepatic level of glucose-6-phosphatase (Figure 5B). These results substantiate the in vivo hypoglycemic activity of fermented SS and reveal that modulation of hepatic glucose metabolic enzyme activities is one of the potential action mechanisms. 

### 2.5. Oral Administation with Fermented SS Attenuated Dyslipidemia in Diabetic Rats

**Figure 6:**
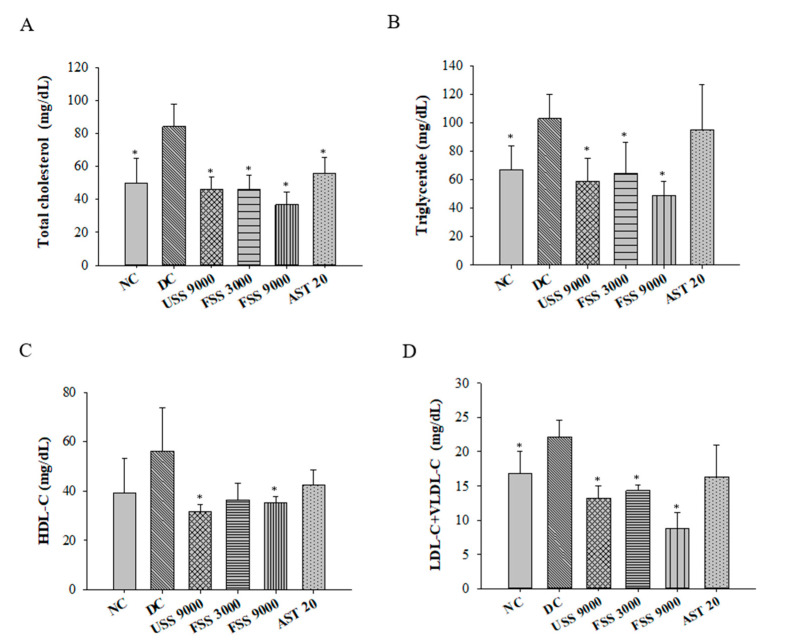
The plasma levels of (**A**) total cholesterol, (**B**) triglycerides, (**C**) HDL-C ^1^, and (**D**) LDL-C ^2^ + VLDL-C ^3^ of rats fed with various diets for 7 weeks. Results are expressed as mean ± SD for each group of rats (N = 6). * *p* < 0.05 compared with diabetic control (DC). ^1^ HDL-C: high-density lipoprotein cholesterol; ^2^ LDL-C: low-density lipoprotein cholesterol; ^3^ VLDL-C: very-low-density lipoprotein cholesterol.

**Figure 7:**
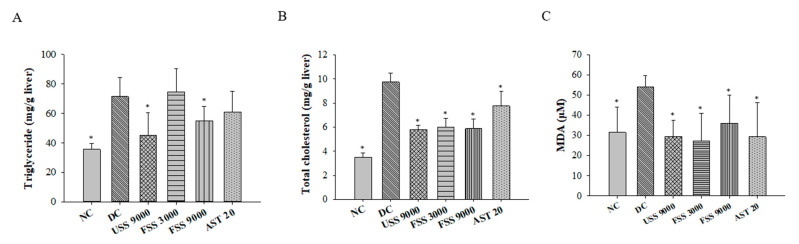
The hepatic levels of (**A**) triglycerides, (**B**) total cholesterol, and (**C**) malondialdehyde (MDA) of rats fed with various diets for 7 weeks. Results are expressed as mean ± SD for each group of rats (N = 6). * *p* < 0.05 compared with diabetic control (DC).

Since dyslipidemia is crucial to induce diabetic complications, the plasma concentrations of TC, TGs, HDL-C, and LDL-C + VLDL-C were further investigated. Compared with normal rats, the plasma TC, TG, and LDL-C + VLDL-C concentrations of the diabetic control rats were significantly increased (Figure 6). However, unfermented SS and fermented SS treatment significantly reduced plasma levels of TC, TGs, and LDL-C + VLDL-C (Figure 6A–C). Interestingly, the decreased HDL-C levels were also observed in rats treated with unfermented SS (9000 mg/kg BW) and fermented SS (9000 mg/kg BW) (Figure 6D). Consistently, both unfermented SS and fermented SS downregulated the levels of hepatic TGs and TC (Figure 7A,B). Most importantly, unfermented SS and fermented SS ameliorated the diabetic increase in hepatic lipid peroxidation evidenced by decreased MDA levels (Figure 7C). 

### 2.6. Oral Administation with Fermented SS Improves Kidney Function in Diabetic Rats

**Figure 8:**
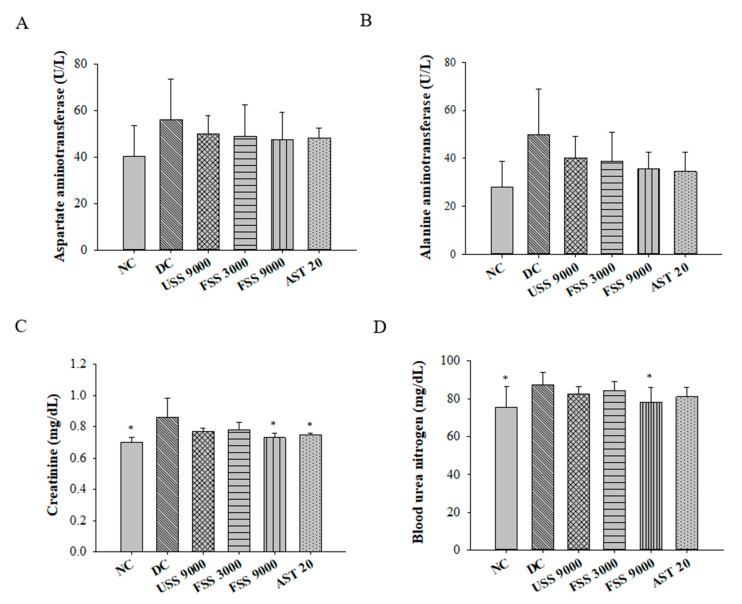
The levels of plasma concentration of (**A**) aspartate amino transferase, (**B**) alanine amino transferase, (**C**) creatinine, and (**D**) blood urea nitrogen of rats fed with various diets for 7 weeks. Results are expressed as mean ± SD for each group of rats (N = 6). * *p* < 0.05 compared with diabetic control (DC).

In diabetic patients, fat accumulation and oxidative stress lead to fatty liver and nephropathy [33]. Therefore, the plasma concentrations of aspartate amino transferase, alanine amino transferase, creatinine, and blood urea nitrogen were determined to preliminarily evaluate the effect of fermented SS on improving diabetic hepatopathy and nephropathy. The levels of aspartate amino transferase and alanine amino transferase were comparable between each group, indicating this model would not result in obvious liver impairment (Figure 8A,B). However, the concentrations of creatinine and blood urea nitrogen were statistically raised in the diabetic rats compared to that in the normal rats (Figure 8C,D). Notably, fermented SS treatment improved renal function in diabetic rats, as evidenced by decreased creatinine and blood urea nitrogen levels (Figure 8C,D). One of the possible mechanisms for ameliorating renal injury is that astaxanthin can protect islet β cells from glucotoxicity and inhibit the production of advanced glycation end products, which lead to renal injury [34].

## 3. Discussion

Type 2 diabetes is a chronic metabolic disease characterized by hyperglycemia and often accompanied by dyslipidemia. Both hyperglycemia and hyperlipidemia can lead to fatal complications. Since medications used to treat type 2 diabetes often cause adverse effects, scientists are very interested in the development of alternative therapies and functional foods. In this study, a process flow for the fermentation of waste SS with mixed strains was established. The in vitro and in vivo experimental results clearly demonstrated the beneficial effects of fermented SS on the improvement of diabetic hyperglycemia and dyslipidemia. In addition, the potential action mechanism of fermented SS was elucidated by analyzing the contents of bioactive components, studying glucose uptake and insulin resistance, and measuring hepatic glucose metabolizing enzyme activities and lipid peroxidation levels.

Due to the practical value of chitin and chitosan, most of the research related to SS reuse is aimed at the extraction of chitin and chitosan [35,36]. Although chemical treatment can rapidly degrade SS to isolate chitin/chitosan, it causes chemical contamination, reduces the biological activity of chitin/chitosan, and rarely harvests other biologically active compounds [37,38]. Alternatively, fermentation of SS with proteolytic bacteria and acid-producing bacteria could separate chitin/chitosan from protein and minerals with less environmental pollution [37,38]. In SS, astaxanthin is usually present in a protein-bound form through non-covalent interactions, which can be disrupted by acid treatment to release astaxanthin [39]. Therefore, it is recommended to harvest components with higher bioactivity from SS by fermentation. In this study, SS was fermented with lactic acid bacteria (LV33,204), proteolytic bacteria (LV2122), and chitinolytic bacteria (LV1111) to obtain bioactive substances with smaller molecular weights, such as astaxanthin, phenolic substances, peptides, amino acids, and chitin derivatives. 

The hypoglycemic potential of SS waste was mentioned in the study of Ketnawa et al., showing that the protease hydrolysate of SS (1 mg/mL) effectively inhibited the activity of the dipeptidyl peptidase-IV inhibitor, suppressed the degradation of GLP-1 and GIP, and subsequently, prolonged the fasting glucose-lowering effect of insulin [40]. Astaxanthin is considered one of the main bioactive components that contributes to the hypoglycemic activity of fermented SS because the protective effect of astaxanthin in the treatment of diabetic hyperglycemia has been demonstrated in animal studies and clinical trials. For example, astaxanthin can improve glucose metabolism by increasing glycogen storage in the liver and promote the translocation of glucose transporter GLUT-4 to the cell membrane for glucose uptake [41,42,43]. Treatment of insulin-resistant rats with astaxanthin (2 mg/kg body weight) for 45 days reduced IRS protein serine phosphorylation, promoted insulin IRS-PI3K-Akt signaling, and improved glucose metabolism by regulating metabolic enzymes [42]. In alloxan-induced diabetic rats, astaxanthin (20 mg/kg body weight) supplementation for 3 weeks significantly reduced fasting blood glucose and cholesterol levels and increased hepatic antioxidant enzyme activity [44]. On the other hand, Hsieh et al. reported that feeding chitosan from shrimp shells to diabetic rats (5% and 7% diet) for 10 weeks significantly reduced plasma glucose and HOMA-IR values [45]. Since the liver is of prime importance in carbohydrate metabolism, this study focused on the activities of hepatic glucose metabolic enzymes. However, the role of skeletal muscle and adipose tissue in the fermented SS-induced hypoglycemic effect needs to be further evaluated, because skeletal muscle and adipose tissue are also involved in the metabolism of glucose [46]. Based on the results of this study, modulation of hepatic glucose metabolic enzyme activity is one of the action mechanisms of fermented SS. As previous studies reported, the effect of astaxanthin on increasing GLUT-4 translocation [41,42,43] and modulating GLUT-4 expression is considered as one of the potential mechanisms of fermented SS, which contains a higher level of astaxanthin than that of unfermented SS. 

Elevated cholesterol absorption and triglyceride synthesis are commonly observed in patients with diabetes, and fat accumulation in the liver and kidneys contributes to fatty liver, kidney disease, and weight gain in liver, kidney, and adipose tissue [16,33,44]. Consistent with previous studies showing the inhibitory effect of chitosan on intestinal lipogenesis and lipid absorption [31,47], fermented SS effectively inhibited weight gain, adipose tissue, liver, and kidney weights, and TC, TGs, HDL-C, and LDL-C without changing food intake. Fermented SS also promotes defecation due to the dietary fiber properties of chitin/chitosan [48]. It has been indicated that the combination of chitosan and bile acids results in direct excretion of bile rather than enterohepatic circulation of bile acids. Therefore, chitosan can promote the conversion of cholesterol into bile acids and relieve hyperlipidemia [49]. In addition to chitosan, 61 patients with mild hyperlipidemia that received 6, 12, and 18 mg astaxanthin daily for 12 weeks showed decreased plasma TG concentrations and increased HDL-C and adiponectin levels, which were involved in the regulation of energy metabolism and the reduction of insulin resistance [50]. Astaxanthin is able to modulate the PPAR-α receptor, a transcriptional base primarily involved in fatty acid metabolism and oxidation, and then is able to reduce the accumulation of cellular lipids [50,51].

Sustained hyperglycemia can cause glycation of proteins, lipids, nucleic acids, and other substances, and excessive glycation can lead to increased oxidative stress, lipid peroxidation, and long-term inflammatory responses [52]. Astaxanthin has been shown to have strong antioxidant capacity against free radical reactions and lipid peroxidation because of its 13 conjugated double bonds and polar hydroxyl groups in its chemical structure [53]. It has been suggested that the protective effect of astaxanthin on diabetes is closely related to its antioxidant activity [34]. In addition to astaxanthin, the antioxidant activity of protein hydrolysates from alcalase hydrolysis of shrimp waste and the extraction of phenolic hydroxyl compounds from SS was reported [22,54]. Chitin and chitosan containing hydroxyl and amine groups that interact with free radicals can protect hepatocytes from oxidative damage [55]. Consistently, the results of this study show the antioxidant potential of fermented SS evidenced by its effect on reducing hepatic MDA levels. 

## 4. Materials and Methods

### 4.1. Shrimp Shells (SSs), Microorganisms, Chemicals, Reagents, and Detection Kits

*Litopenaeus vannamei* shrimp shells were harvested from the Kanzaiding fish market (Keelung, Taiwan). Lactic acid bacteria (LV33204), proteolytic bacteria (LV2122), and chitin-decomposing bacteria (LV1111) were isolated from *Litopenaeus vannamei* and identified as *Lactobacillus plantarum*, *Stenotrophomonas maltophilia*, and *Aeromonas dhakensis*, respectively, by the Food Industry Research and Development Institute (Hsinchu, Taiwan). Unless otherwise stated, the chemicals and reagents were purchased from Sigma-Aldrich Chemical Co. (St. Louis, MO, USA), Merck Ltd. (Darmstadt, Germany), and Kanto Chemical Co. (Tokyo, Japan). Reagents and broth for bacterial culture were purchased from Difco Laboratories Inc. (Detroit, MI, USA). Kits for detection of cholesterol and TGs were purchased from Randox Laboratories Limited (Crumlin, UK).

### 4.2. Fermentation of SS by Mixed Strains

The strains of *S. maltophilia* LV2122 and *A. dhakensis* LV1111 were stored in glycerol-containing nutrient broth, whereas the strain of *L. plantarum* LV33204 was kept in glycerol-containing MRS medium at −80 °C. Before inoculation, all strains were subcultured twice in fresh medium without glycerol at 37 °C for 24 h.

After removal of the residual meat, the SS was washed with plenty of running water, drained, and lyophilized. The lyophilized SS was ground and sieved with a 60-mesh sieve. In a 1 L flask, 40 g of SS powder was mixed with 360 mL of deionized water. After sterilization at 121 °C for 20 min and cooling, the sterilized SS was inoculated with LV1111 and LV2122 cultures (to make both the initial bacterial count of 10^5^ CFU/mL), and incubated at 37 °C with shaking at 150 rpm for 2 days. Then, glucose (1% *w*/*w*) and LV33204 culture (to make the initial count of 10^5^ CFU/mL) were added, and incubated at 37 °C without shaking for another 5 days. The fermented and unfermented SS products were lyophilized and kept at −20 °C. 

On the other hand, 2 g of lyophilized powders of fermented and unfermented SS products were added with 20 mL of methanol and sonicated at room temperature for 15 min. After centrifugation, the supernatants were dried with nitrogen. The residues were dissolved in 0.1% dimethyl sulfoxide to prepare the stock solution (2 mg/mL) for cell culture experiments.

### 4.3. Determination of Crude Protein, Ash, Total Phenolic Content, Total Peptides, and Astaxanthin Contents within Fermented SS and Amino Acid Analysis

The proximate composition of unfermented and fermented SS was analyzed following the Association of Official Analytical Chemists (AOAC) official methods of analysis [56]. To determine the content of total peptides, diluted samples were centrifuged at 10,000× *g* for 10 min, and 25 μL of the supernatant was mixed with 1 mL of OPA reagent and kept in the dark for 2 min. Absorbance was measured at 340 nm, and leucine-glycine was employed as a standard [57]. Total soluble phenolic contents in the samples were determined with the Folin–Ciocalteu reagent and gallic acid was used as a standard phenolic compound [58]. Briefly, 1 mL of supernatant of fermented SS broth was mixed with 9 mL distilled water. After being added with 1 mL of Folin–Ciocalteu reagent and mixed thoroughly, 3 mL of Na_2_CO_3_ (2%) was added, and the mixture was incubated for 2 h with intermittent shaking. The absorbance at 760 nm was measured. According to the method established in the previous study [44], the samples were extracted by butylated hydroxytoluene (0.1 mg/mL)-containing acetone. The amount of astaxanthin in the extraction was analyzed by high-performance liquid chromatography using a C18 reversed phase (250 mm × 4.6 mm) along with an RID detector. Quantitative amino acid analysis was conducted using a Beckman System 6300 amino acid analyzer (Beckman Instruments, Inc., Brea, CA, USA). 

### 4.4. Effect of Fermented SS on Glucose Uptake of Insulin Resistant FL83B Hepatocytes

FL83B cells, supplied by the Bioresource Collection and Research Center (BCRC; Hsinchu, Taiwan), were cultured in F12K medium containing 10% FBS and 1% penicillin and streptomycin mix (Invitrogen Corp., Camarillo, CA, USA) at 37 °C in a 5% CO_2_ environment. To investigate the cytotoxicity of tested samples, FL83B cells (1 × 10^5^ cells/mL) were incubated in the presence of various samples for 24 h. A 3-(4,5-dimethylthiazol-2-yl)-2,5-diphenyl-tetrazolium bromide (MTT) solution was added to each well (1 mg/mL) and incubated for 4 h. At the end of incubation, the formed formazan was dissolved with dimethyl sulfoxide, and then the absorbance at 570 nm was measured. Cell viability (%) = (absorbance of cells treated with test sample/absorbance of cells treated without test sample) × 100%. Insulin resistance of FL83B cells was induced by the method described by Huang et al. [59]. Briefly, FL83B cells were seeded in 10 cm dishes and incubated at 37 °C to reach 80% confluence. Serum-free F12K medium containing 40 ng/mL of recombinant mouse TNF-α was added and incubated for 5 h to induce insulin resistance. Subsequently, FL83B cells were suspended in HBSS containing 5 mM glucose and 10 ng/mL insulin. The cell suspensions were incubated with the test samples for 24 h. The glucose concentration in the supernatants was determined by a glucose detection kit (Audit Diagnostics, Cork, Ireland) following the manufacturer′s instructions. Glucose uptake of FL83B cells (%) = (the original concentration of glucose − the concentration of glucose in the cultured supernatants)/the original concentration of glucose × 100%. All experiments were performed in triplicate.

### 4.5. Animals and Experimental Design 

Seven-week-old male Sprague Dawley rats from BioLASCO Taiwan Co., Ltd. (Taipei, Taiwan) were housed in the terrestrial animal experimental room of National Taiwan Ocean University under 30–70% of relative humidity, 21–25 °C temperature conditions, and a 12 h light/dark cycle. All animal experiments were conducted following the guidelines for the care and use of laboratory animals and approved by the Institutional Animal Care and Use Committee of the National Taiwan Ocean University (NTOU-109,072). After 1 week of adaption, the rats were randomly divided into 6 groups (N = 6): normal control (NC), diabetic control (DC), and diabetic rats fed with unfermented SS (9000 mg/kg body weight; USS9000), with fermented SS (3000 and 9000 mg/kg body weight; FSS3000 and FSS9000), or with astaxanthin (20 mg/kg body weight; AST20). The preparation and administration of streptozotocin (STZ) and nicotinamide (NA) to induce type 2 diabetes was in accordance with the methods reported in previous studies [60,61,62]. Rats with a body weight over 300 g were subcutaneously injected with fresh STZ (65 mg/kg BW) in 0.01 M citrate buffer (pH = 4.6), whereas nicotinamide (NA) was dissolved in saline and administered subcutaneously (230 mg/kg BW) 15 min before STZ. After 7 days, the OGTT was performed to affirm the successful induction of diabetes, and the diabetic rats were fed with unfermented or fermented SS, or astaxanthin supplement daily for 7 weeks. The intake of feed and water, body weight, urine volume, and stool weight were recorded weekly. The OGTT was performed again 7 weeks after induction of diabetes, and the rats were sacrificed 3 days after the most recent OGTT to harvest blood, liver, kidney, and intestinal tissues for further analysis.

### 4.6. Measurement of Plasma Levels of Glucose, Insulin, Aspartate Amino Transferase, Alanine Amino Transferase, Creatinine, and Blood Urea Nitrogen

Blood samples were harvested from individual rats at 0, 30, 60, 90, and 120 min after the beginning of the OGTT and before sacrifice to prepare plasma. The plasma levels of glucose, insulin, aspartate amino transferase, alanine amino transferase, creatinine, and blood urea nitrogen were determined by a glucose detection kit, an insulin ELISA kit, and enzymatic kits (Randox Laboratories Limited, Crumlin, UK), respectively, following the manufacturer′s instructions. The equation for the HOMA-IR calculation was according to that reported in a previous study [3].

### 4.7. Plasma Levels of TGs, TC, and Lipoprotein Cholesterol Determination

Before animal sacrifice, blood samples were harvested individually to obtain plasma. The plasma levels of TGs and TC were determined by enzymatic kits (Randox Laboratories Limited) following the manufacturer′s instructions. LDL and HDL were isolated by using a modified phosphotungstic acid/magnesium chloride precipitation procedure described in a previous study [63]. The levels of cholesterol in the HDL-containing supernatants and LDL-containing precipitations were determined by enzymatic kits (Randox Laboratories Limited) following the manufacturer′s instructions. 

### 4.8. Determination of TGs and TC in the Liver Tissues 

After animal sacrifice, the liver tissues were isolated and homogenized (SA-50 Max. 3000; Hong Sheng, Taipei, Taiwan) in chloroform/methanol solution for 1 min. After centrifugation, the supernatants were mixed with Triton X-100. To remove the solvent, vacuum evaporation (SC 110; Savant Instruments, Holbrook, NY, USA) was employed to harvest lipids [64]. The levels of TGs and TC were determined by enzymatic kits (Randox Laboratories Limited) following the manufacturer′s instructions.

### 4.9. Determination of Hexokinase, Glucose-6-Phosphatase, and Glucose-6-Phosphate Dehydrogenase in the Liver

After animal sacrifice, the liver tissues were isolated and homogenized in N-acetyl-cysteine buffer. After centrifugation, the cytosol of hepatocytes was harvested, and hexokinase, glucose-6-phosphatase, and glucose-6-phosphate dehydrogenase activities in the cytosol were determined following the method described in the previous study [65].

### 4.10. Determination of Malondialdehyde (MDA) Levels in the Liver

Based on the protocol of a previous study [66], the liver tissues were homogenized in 1.15% KCl solution and then mixed with 2-thiobarbitutric acid. The mixtures were incubated in the boiled water bath for 45 min, followed by a cooling down period. After centrifugation at 1600× *g*, 4 °C for 10 min, the collected supernatants were incubated at room temperature for 30 min, and their MDA levels were detected by a Luminescence Spectrometer (Hitachi, F2000, Tokyo, Japan) with excitation at 520 nm and emission at 535 nm.

### 4.11. Statistical Analysis

The data were analyzed by using SPSS Version 12.0 (SPSS Inc., Chicago, IL, USA). Statistical differences between each group and multiple comparisons of means were analyzed by one-way analysis of variance (ANOVA) and Duncan′s multiple range test, respectively. Statistical significance was set at *p* < 0.05. The results are expressed as mean ± standard deviation.

## 5. Conclusions

In this study, the process for SS waste by fermentation with mixed strains was established. After fermentation, the contents of bioactive components beneficial for improving diabetes were increased. The results of in vitro and in vivo experiments substantiate the protective effects and potential mechanism of fermented SS against diabetic hyperglycemia, hyperlipidemia, lipid peroxidation, and kidney dysfunction. Although further studies are required to comprehensively understand the cellular and molecular mechanism of fermented SS, this is the first study demonstrating that the fermented SS has the potential to be developed into functional foods for the management of diabetes.

## Figures and Tables

**Table 1:** Free amino acid compositions in the supernatant of unfermented and fermented SS.

Free Amino Acids	Unfermented (mg/100 g)	Fermented (mg/100 g)
Phosphorserine	2.20	2.99
Taurine	2.05	2.29
Aspartic Acid	3.59	3.52
Threonine	1.60	9.74
Serine	2.39	1.03
Asparagine	0.68	N.D.
Glutamic Acid	3.88	27.91
L-2-Aminoadipic Acid	N.D.	3.31
Glycine	5.26	16.13
Alanine	2.79	58.66
Citrulline	N.D.	12.73
Valine	2.58	38.79
Methionine	N.D.	4.58
Isoleucine	1.28	18.12
Leucine	2.49	21.49
Tyrosine	4.95	30.52
Phenylalanine	N.D.	26.49
Γ-Aminobutyric Acid	0.11	1.25
Ethanolamine	0.33	1.24
Dl-Plus Allo-Δ-Hydroxylysine	0.10	0.66
Ornithine	0.85	5.95
Lysine	3.19	N.D.
Histidine	N.D.	3.40
Arginine	6.11.	N.D.
Hydroxyproline	N.D.	1.80
Proline	N.D.	17.19
**Total free amino acids**	46.52	309.79

N.D. means not detected.

## Data Availability

The datasets analyzed in the current study are available from the corresponding author upon request.
